# Histone acetyltransferases CBP/p300 in tumorigenesis and CBP/p300 inhibitors as promising novel anticancer agents

**DOI:** 10.7150/thno.73223

**Published:** 2022-06-21

**Authors:** Qingjuan Chen, Binhui Yang, Xiaochen Liu, Xu D. Zhang, Lirong Zhang, Tao Liu

**Affiliations:** 1 Department of Oncology, 3201 Hospital of Xi'an Jiaotong University Health Science Center, Hanzhong, Shaanxi 723000, China.; 2 School of Medicine and Public Health, Priority Research Centre for Cancer Research, University of Newcastle, Callaghan, Newcastle, NSW 2308, Australia.; 3 Translational Research Institute, Henan Provincial People's Hospital, Academy of Medical Sciences, Zhengzhou University, Zhengzhou, China.; 4 Department of Pharmacology, School of Basic Medical Sciences, Zhengzhou University, Zhengzhou, China.; 5 Children's Cancer Institute Australia, Randwick, Sydney, NSW 2031, Australia.; 6 School of Women's and Children's Health, University of New South Wales, Sydney, New South Wales, Australia.

**Keywords:** CBP/p300, gene transcription, tumorigenesis, small molecule inhibitors, cancer therapy

## Abstract

The histone acetyltransferases CBP and p300, often referred to as CBP/p300 due to their sequence homology and functional overlap and co-operation, are emerging as critical drivers of oncogenesis in the past several years. CBP/p300 induces histone H3 lysine 27 acetylation (H3K27ac) at target gene promoters, enhancers and super-enhancers, thereby activating gene transcription. While earlier studies indicate that CBP/p300 deletion/loss can promote tumorigenesis, CBP/p300 have more recently been shown to be over-expressed in cancer cells and drug-resistant cancer cells, activate oncogene transcription and induce cancer cell proliferation, survival, tumorigenesis, metastasis, immune evasion and drug-resistance. Small molecule CBP/p300 histone acetyltransferase inhibitors, bromodomain inhibitors, CBP/p300 and BET bromodomain dual inhibitors and p300 protein degraders have recently been discovered. The CBP/p300 inhibitors and degraders reduce H3K27ac, down-regulate oncogene transcription, induce cancer cell growth inhibition and cell death, activate immune response, overcome drug resistance and suppress tumor progression *in vivo*. In addition, CBP/p300 inhibitors enhance the anticancer efficacy of chemotherapy, radiotherapy and epigenetic anticancer agents, including BET bromodomain inhibitors; and the combination therapies exert substantial anticancer effects in mouse models of human cancers including drug-resistant cancers. Currently, two CBP/p300 inhibitors are under clinical evaluation in patients with advanced and drug-resistant solid tumors or hematological malignancies. In summary, CBP/p300 have recently been identified as critical tumorigenic drivers, and CBP/p300 inhibitors and protein degraders are emerging as promising novel anticancer agents for clinical translation.

## Introduction

Gene transcription is carried out by RNA polymerase (RNA Pol) enzymes, and RNA Pol II is responsible for the synthesis of mRNAs and non-coding RNAs 1. To start gene transcription, RNA Pol II recognizes the promoter region of the target gene, synthesizes RNA from the DNA duplex, and then moves from the promoter to the gene body (elongation) until RNA synthesis completes at a termination signal 2, 3.

Transcriptional enhancers are short DNA elements which are characterized by acetylated histone H3 lysine 27 (H3K27ac) signals. Transcriptional enhancers bind RNA Pol II, transcription factors and co-regulators to enhance the transcription of neighboring genes, independent of their orientation, as enhancers can loop over long genomic distances to regulate promoter activity 4. First reported in 2013, super-enhancers span tens of kilobases of DNA sequences, consist of clusters of enhancers, and selectively localize at cell identity gene and critical oncogene loci 5, 6. Characterized by massive H3K27ac signals, super-enhancers are densely bound by master transcription factors, co-regulators and mediators 6-10.

The histone acetyltransferase CREB-binding protein (CBP) and its closely related p300 protein are often referred to as a single entity (CBP/p300), due to their considerable sequence homology and functional overlap and co-operation 11. CBP/p300 are required for histone acetylation globally 11-14, at promoters 15, enhancers 12, 13 and super-enhancers 16. Known as tumor suppressors in certain cancers more than a decade ago 17-19, CBP/p300 have recently been demonstrated as important regulators of enhancer- and super-enhancer-mediated transcriptional activation of critical oncogenes 13, 16, 20, and small molecule CBP/p300 inhibitors are emerging as efficacious anticancer agents 13, 20, 21.

## CBP/p300 protein structure and histone acetyltransferase activity

**Figure 1:**
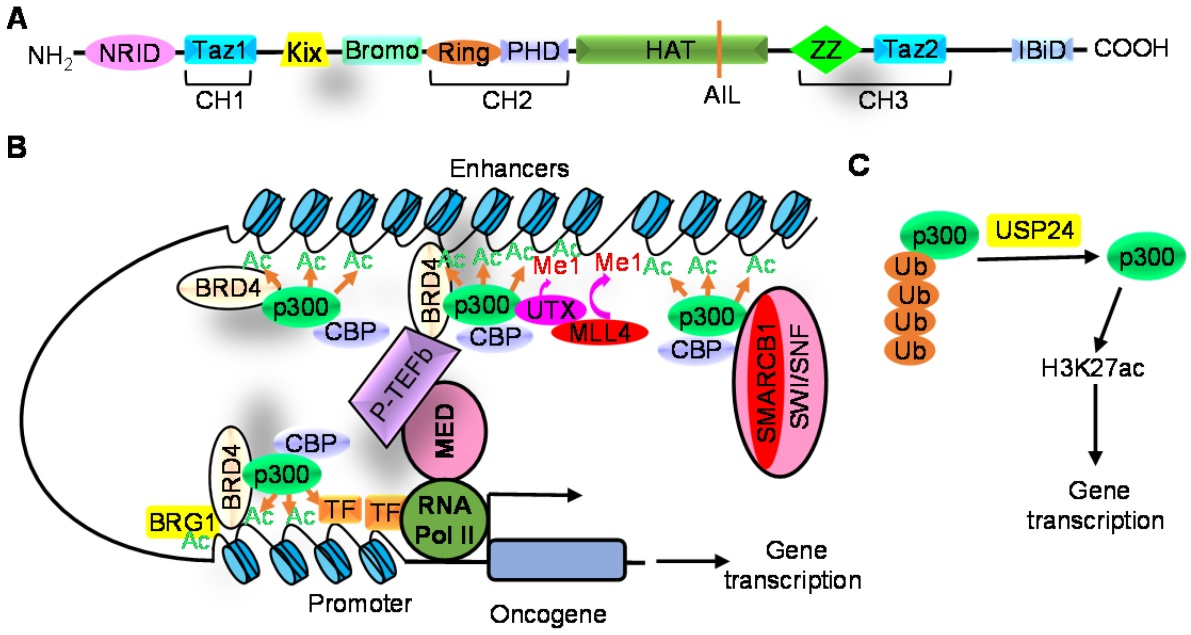
Modulation of CBP/p300-induced histone acetylation by histone reader, histone modification proteins and the ubiquitin hydrolase USP24. **A.** CBP/p300 proteins consist of nuclear receptor interaction domain (NRID), transcriptional-adaptor zinc-finger domain 1 (TAZ1, also known as CH1), kinase inducible domain of CREB interacting domain (KIX), bromodomain (Bromo), PHD finger, histone acetyltransferase domain (HAT) including the autoinhibitory loop (AIL), ZZ-type zinc finger domain (ZZ), TAZ2 and interferon-binding domain (IBiD). **B.** CBP/p300 interact with BRD4 to induce H3K27ac, and BRG1 is then recruited to acetylated histone sites to enhance H3K27ac and gene transcription. P300 also forms a complex with UTX and MLL4, driving H3K4 mono-methylation (Me1) which further augments H3K27ac and transcriptional activation. In addition, SMARCB1 and other SWI/SNF subunit proteins recruit p300 to distal enhancers, rather than promoters, to induce H3K27ac and gene transcription. **C.** USP24 decreases p300 protein ubiquitination and proteasome-mediated degradation, thereby increasing p300 protein expression and H3K27ac.

CBP/P300 proteins have intrinsic histone acetyltransferase (HAT) activity due to their HAT domains and, like CBP/p300 associated factor (PCAF), induces the acetylation of histone H3 and H4 14, 22-24. The catalytic core of CBP/p300 proteins consists of the bromodomain, HAT, cysteine/histidine-rich region (CH2, comprising PHD and RING) and ZZ-type zinc finger (ZZ) domains (Figure 1A). The bromodomain, HAT and PHD domains establish a structural unit to which the RING and ZZ domains connect 25-27. The catalytic core is preceded by the N-terminal nuclear receptor interaction domain (NRID), the transcriptional adaptor zinc-finger 1 (TAZ1) and the kinase-inducible CREB interaction region (KIX); and is succeeded by the second TAZ domain (TAZ2) and the interferon-binding domain (IBiD) 28-30 (Figure 1A). Identical mutations in the HAT domains of p300 and CBP reduce acetyltransferase activity to different extents, indicating minor differences between CBP and p300 proteins 31.

The bromodomain/PHD finger region of CBP/p300 can bind to acetylated HAT domain of CBP/p300 and magnify HAT activity. The binding of the bromodomain to the acetyl-lysine is required for H3 and H4 lysine acetylation 13, 26, 32, inhibition of the bromodomain leads to histone deacetylation 13, and deletion of the PHD finger reduces histone acetylation 33. The ZZ domain recognizes histone H3 tail and promotes acetylation of histone H3K27 and H3K18 sites 26.

The RING finger of CBP/p300 suppresses HAT activity through blocking the HAT active site 25, and the autoinhibitory loop (AIL) of the HAT domain, when in a hypoacetylated form, also suppresses HAT activity 34-36. Several lysine residues in the AIL are autoacetylated by the HAT domain, and autoacetylated AIL binds intramolecularly to the bromodomain and facilitates histone H3 acetylation 32, 37.

Interestingly, p300 has been found to form phase-separated condensates in the nucleus, in a HAT domain-dependent manner, and the p300 condensates bind to histone H3 tail but show reduced HAT activity 38. The p300 condensates at repressed chromatin regions therefore function as a storage pool of p300 with reduced HAT activity 38.

## Modulation of CBP/p300-induced histone acetylation

Histone acetylation and consequent transcriptional regulation by CBP/p300 is modulated by the histone “reader” BET bromodomain protein BRD4 39, histone modification proteins 34, 40-42 and transcription factors 36. CBP/p300 interacts with BRD4 to induce H3K27ac, and BRG1 is then recruited to acetylated histone sites 39. In partnership with BRD4 and BRG1, CBP/p300 play an important role in inducing H3K27ac and the transcription of pluripotency genes, such as OCT4 and NANOG, in embryonic stem cells 39 (Figure 1B).

Drosophila Polycomb and its mammalian counterpart CBX proteins directly interact with un-acetylated CBP HAT domain at its autoinhibitory loop and suppresses HAT activity and H3K27ac at gene promoters and enhancers 34. CBP autoacetylation inhibits Polycomb/CBX binding and augments H3K27ac 34. The SET domains of Trithorax and Trithorax-related proteins show substantial histone H3K4 mono-methyltransferase activity. CBP, Trithorax and Trithorax-related proteins form complexes, and H3K4 mono-methylation enhances CBP-mediated H3K27ac at transcriptional enhancers 40.

P300 also forms an epigenetic protein complex with the H3K27 demethylase UTX and the H3K4 methyltransferase MLL4. UTX facilitates p300-modulated H3K27ac through recruiting p300 to enhancer and super-enhancer regions; p300, UTX and MLL4 form a feedforward regulatory loop that drives simultaneous H3K4 mono-methylation and H3K27ac on enhancers and super-enhancers to generate an active enhancer landscape; and MLL4-dependent H3K4 mono-methylation further augments CBP/p300-dependent H3K27ac and transcriptional activation 41, 42. Through forming a protein complex with p300, the SMARCA4, SMARCB1 and SMARCC1 SWI/SNF subunit proteins recruit p300 to distal enhancers, rather than promoters, to induce H3K27ac and enhancer-associated gene transcription 43 (Figure 1B).

Transcription factors stimulate HAT activity, as CBP protein recruited by wild-type, but not dominant-negative mutant, transcription factors shows strong HAT activity 44. The double homeodomain transcription factor DUX4 interacts with p300/CBP and recruits CBP/p300 to its target genes to induces H3K27ac and gene transcription 45. Transcription factor ligands such as IRF3 and STAT1 bind to p300, and IRF3 and STAT1 dimerization renders p300 trans-autoacetylation in the HAT domain which augments p300 HAT activity 36.

CBP/p300 HAT activity is augmented by phosphorylation 46, 47, de-ubiquitination 15 and enhancer RNAs 48. DYRK1A and mTORC1 induce p300 protein phosphorylation which blocks its RING domain from binding to its HAT domain, resulting in suppression of intra-molecular HAT activity inhibition 46, 47. The ubiquitin hydrolase USP24 directly interacts with p300 protein, and reduces p300 protein ubiquitination and proteasome-mediated degradation, leading to p300 protein up-regulation and histone acetylation 15 (Figure 1C).While CBP is highly enriched at enhancers, enhancer RNAs bind to the RNA-binding region of the CBP HAT domain, resulting in improved substrate binding, magnified HAT activity and up-regulated transcription and expression of neighbouring protein-coding genes 48.

## CBP/p300 are required for promoter-, typical enhancer- and super-enhancer-driven gene transcription

CBP/p300 can modulate gene transcription, directly through acetylating histone at gene promoters, typical enhancers and super-enhancers 39, 49-53 or indirectly through inducing the acetylation of transcription factors, such as p53, MYC and GATA-1 54-56. CBP/p300 are required for histone acetylation at BRD4-occupied sites and for BRD4 binding at gene promoters and enhancers 57 (Figure 1B). The recruitment of BRD4 by CBP/p300 in turn recruits and activates RNA Pol II and positive transcription elongation factor b (P-TEFb), leading to transcriptional initiation and the release of RNA Pol II from promoter-proximal-pause for elongation 16. CBP/p300 can also recruit RNA Pol II, independent of BRD4, and stimulate transcription factor IID binding and pre-initiation complex assembly at gene promoters, enhancers and super-enhancers to activate gene transcription 16.

CBP/p300 bromodomain inhibition results in H3K27 deacetylation and reduction in enhancer-associated gene expression 13. In comparison, CBP/p300 HAT inhibition down-regulates gene transcription, similar to that of CBP/p300 knockout, suggesting that CBP/p300 HAT activity is the main factor for CBP/p300-dependent gene transcription 58.

CBP/p300 play a key role in maintaining cell identity-specific gene expression and therefore cell identity 59. In embryonic stem cells, the histone variant H3.3 is enriched at enhancers 60. When phosphorylated, H3.3 stimulates p300 HAT activity to result in H3K27ac at gene enhancers and gene transcription 60. Inhibition of CBP/p300 HAT activity induces global histone de-acetylation and prevents pluripotent stem cell formation, indicating that HAT activity is required for the function of a number of transcription factors and for reprogramming 59.

In genome- and proteome-wide analyses, CBP/p300 have been found to bind to and acetylate > 200 histone modification proteins, transcription factors and transcriptional co-activators, thereby regulating gene transcription 12. For example, CBP/p300 bind to and acetylate the sequence-specific DNA-binding sites of p53, c-Myc and GATA-1 proteins to enhance their DNA-binding activity 54-56. Acetylation of p53 by CBP/p300 leads to p53 protein conformational change and increased transcription of p53 target genes such as p21 and MDM2 54, 61, 62, acetylation of GATA-1 protein by p300 stimulates GATA-1-mediated gene transcription 55, and acetylation of c-Myc protein at its carboxy-terminal by CBP augments c-Myc target gene transcription 56.

## CBP/p300 as tumor suppressors

Reduction in CBP expression due to chromosomal rearrangement, chromosomal deletion or point mutation results in Rubenstein-Taybi Syndrome, and patients are prone to develop tumors 63. In a mouse model of human myelodysplastic syndrome, deletion of *EP300* significantly accelerates leukemogenesis. Mechanistically, *EP300* deletion activates the mitogen-activated protein (MAP) kinase and the Janus kinase (JAK)/signal transducer and activator of transcription (STAT) pathways, suppresses apoptosis and restores hematopoietic progenitor and stem cell self-renewal 64. In chimeric mice, loss of CBP or p300 leads to up-regulation of *NOTCH1*, *BMI1*, *MYC*, *CCNE* and *SKP2* oncogenes, as well as the development of thymic lymphoma and histiocytic sarcomas 17-19, further confirming the role of CBP/p300 deletion/loss in tumorigenesis.

Chromosomal rearrangements of the *ZNF384* gene with *CBP* and *EP300* genes result in *CBP-ZNF384* and *EP300-ZNF384* fusion genes and proteins 65. The fusion proteins show loss of CBP/p300 HAT activity in a dominant-negative manner and significantly up-regulate *CLCF1* and *BTLA* gene expression, leading to reduction in hematopoietic stem and progenitor cell differentiation and induction of malignant transformation 65.

## CBP/p300 induce oncogene transcription, cancer cell proliferation, survival, tumorigenesis, metastasis and immune evasion

Transcription landscape is substantially reprogrammed during tumorigenesis. *EP300* gene is amplified or gained in approximately 24% of human hepatocellular carcinoma tissues, and *EP300* gene over-expression positively correlates with the gene copy number variations and poor patient prognosis 66. P300 substantially reprograms super-enhancers during hepatocellular carcinoma tumorigenesis; stimulates over-expression of super-enhancer-associated oncogenes such as *MYC*, *MYCN* and *CCND1*; and induces hepatocellular carcinoma cell proliferation *in vitro* and tumor progression *in vivo* 66 (Table 1).

RNA sequencing data from primary and metastatic castration-resistant prostate cancer tissues show that CBP and p300 mRNAs are over-expressed in tumor than normal tissues, and that CBP and p300 expression positively correlates with androgen receptor expression, androgen receptor signature, and androgen deprivation therapy resistance 67. In castration-resistant prostate cancer cells, CBP and p300 up-regulate androgen receptor signaling by binding to androgen receptor-binding sites at target gene promoters, activate the transcription of oncogenes such as *MYC*, and induce cancer cell proliferation 67 (Table 1).

In melanoma cells, 33 of the 250 genes down-regulated after p300 knockdown are target genes of the transcription factor MITF. P300 up-regulates *MITF* gene expression by inducing H3K27ac at the *MITF* gene promoter, resulting in the up-regulation of MITF target oncogenes, such as *FOXM1*, and melanoma cell proliferation 68. In clear cell renal cell carcinoma, loss of the tumor suppressor *VHL* drives the recruitment of p300 to oncogene enhancers and super-enhancers. P300 thereby induces H3K27ac, *MYC* and *ZNF395* oncogene over-expression, clear cell renal cell carcinoma cell proliferation, survival and colony formation *in vitro*, and tumor progression in mouse models 69 (Table 1).

In T-cell acute lymphoblastic leukemia patients, heterozygous somatic mutations introduce MYB binding motifs upstream of the *TAL1* oncogene 70. MYB binding results in the recruitment of CBP, leading to super-enhancer formation, *TAL1* over-expression, cell survival and leukemogenesis 70 (Table 1). In acute myeloid leukemia, CBP/p300 modulate the transcription of genes involved in DNA replication, DNA repair, mitosis and cell cycle progression. CBP/p300 are therefore essential for acute myeloid leukemia cell proliferation, immortalization, leukemia initiation and maintenance 57, 71. In chronic myeloid leukemia and lymphoma cells, the *GATA1* and *MYC* gene loci are characterized by super-enhancers occupied by p300 72. CBP/p300 bromodomain inhibition blocks *GATA1* and *MYC* oncogene expression and induces chronic myeloid leukemia and lymphoma cell cycle arrest and growth inhibition 72. In diffuse large B-cell lymphoma cells with C-terminal truncated HAT domain-deficient p300, the truncated p300 suppresses NF-κB and REL activity, reduces p53 expression, and is required for lymphoma cell proliferation 37, 73 (Table 1).

In *CBP*-deficient lung cancer cells, genome-wide gene expression analysis identifies *MYC* as one of the top genes most significantly up-regulated by p300, and p300 is essential for CBP-deficient lung cancer cell proliferation and tumor progression *in vivo* 74. In non-small cell lung cancer, p300 up-regulates the transcription of IL-6 in macrophages and cancer cells, increases the expression of mesenchymal markers and decreases the expression of epithelial markers. P300 thereby promotes cancer cell migration, invasion and metastasis 15, 75 (Table 1).

In myelodysplastic syndrome-derived acute myeloid leukemia cells, CBP/p300 promote the expression of ribosomal genes and CBP/p300 inhibition reduces global protein synthesis, leading to leukemia cell death 76.

The recruitment of T regulatory cells and myeloid-derived suppressor cells to tumor tissues is important for immune evasion. CBP/p300 induce H3K27 acetylation at the promoters and enhancers of genes critical for T regulatory cells and myeloid-derived suppressor cells, such as STAT pathway genes, *FOXP3* and *GATA3*, and up-regulate their expression. CBP/p300 thereby augment T regulatory cell and myeloid-derived suppressor cell survival and function; suppress cytotoxic T cell-driven immunity, lymphocyte activation and proliferation; and promote tumor progression *in vivo* 77-80 (Table 1).

## Small molecule compound CBP/p300 activators and inhibitors

### CBP/p300 activators

Small molecule compound CBP/p300 activators and inhibitors have been discovered in the past two decades. CTPB, an amide derivative of anacardic acid, alters p300 protein structure, activates p300 HAT activity, and enhances HAT-dependent transcriptional activation 81. The short-chain fatty acids butyrate and propionate have long been presumed to inhibit histone deacetylases, however, it has recently been shown that butyrate and propionate are converted to acetyl coenzyme A which catalyzes auto-acetylation of the autoinhibitory loop of p300, leading to HAT activation and histone acetylation 82. The findings challenge many other publications demonstrating that butyrate acts mainly by inhibiting histone deacetylases 83, 84, and require independent validation.

### CBP/p300 HAT inhibitors

Anacardic acid from cashew nutshell and Garcinol from garcinia are weak natural inhibitors of both p300 and PCAF HAT activity 81, 85. The coenzyme A conjugate Lys-CoA is an effective p300 HAT inhibitor (half-maximal inhibitory concentration, IC_50_ of 0.5µM) and a much weaker inhibitor of PCAF (IC_50_ of 200µM) 86. The isothiazolone-based PCAF and p300 HAT co-inhibitors CCT077791 and CCT077792 reduce histone acetylation and reduce colon cancer cell proliferation by 50% 72 hours after treatment with CCT077791 at 2-3 µM and CCT077792 at 0.4 µM 87. Through compound screening and medicinal chemistry, “compound 12” was found to selectively suppress CBP/p300 HAT activity with an IC_50_ of 620 nM. “Compound 12” recapitulates p300 siRNA-mediated reduction in estrogen receptor target gene transcription in breast cancer cells 88.

Through *in silico* drug discovery pipelines, highly selective and potent drug-like small-molecule compound CBP/p300 HAT inhibitors have been discovered. The pyrazolone-containing p300 HAT inhibitor C646 suppresses p300-induced HAT activity with an inhibitory constant of 400 nM 89 (Table 2).

Through virtual ligand screen, A-485 has been found to be a potent, selective and drug-like CBP and p300 HAT inhibitor 90. A-485 binds to CBP/p300 catalytic active site, and reduces histone acetylation by CBP and p300 with IC_50_ of 2.6 nM and 9.8 nM respectively 90 (Table 2).

B026 and B029-2, which have similar chemical structure, are very efficient in suppressing CBP (IC_50_ of 9.5nM for B026 and 11nM for B029-2) and p300 (IC_50_ of 1.8nM for B026 and 0.5nM for B029-2) 91, 92 HAT activity (Table 2).

### CBP/p300 bromodomain inhibitors

The CBP/p300 bromodomain inhibitors CPI703 and CPI644 exhibit good efficacy in inhibiting CBP bromodomain with IC_50_ values of 0.47 μM and 0.18 μM respectively and cellular EC_50_ of 2.1 μM and 0.53 μM respectively 93. Treatment with CPI703 or CPI644 reduces H3K27ac and the transcription of a number of genes including *FOXP3* in regulatory T cells, suppresses regulatory T cell differentiation and T helper 17 cell cytokine production 93. The CBP/p300 bromodomain inhibitor CBP30 suppresses CBP (IC_50_ = 21 nM) and p300 (IC_50_ = 38 nM), and reduces IL-17A expression in immune cells and secretion by T helper 17 cells 94 (Table 3).

I-CBP112 is a selective CBP/p300 bromodomain ligand and inhibitor 95, and shows dissociation constant of 151 ± 6 nM for CBP and 167 ± 8 nM for p300, and IC_50_ of 142 nM for CBP and 625 nM for p300 96 (Table 3).

The chemically synthesized CBP/p300 bromodomain inhibitor GNE-049 displays effective suppression of both CBP (IC_50_ = 1.1 nM) and p300 (IC_50_ = 2.3 nM), and exhibits strong efficacy in repressing the expression of oncogenes, such as *MYC* (EC_50_ = 14 nM), in leukaemia cells 97. The structurally similar GNE-781 displays effective suppression of both CBP (IC_50_ = 0.94 nM) and p300 (IC_50_ = 1.2 nM), and strong efficacy in repressing the *MYC* oncogene (EC_50_ = 6.6 nM) in leukaemia cells 98 (Table 3).

CCS1477 is a potent, selective and orally bioavailable CBP/p300 bromodomain inhibitor. CCS1477 binds to CBP and p300 with dissociation constants of 1.7 nM and 1.3 nM respectively and an IC_50_ of 19 nM 67 (Table 3).

### Proteolysis-targeted-chimaera (PROTAC) p300 protein degrader JQAD1

PROTACs are small molecule compounds that target proteins for E3 ligase-mediated ubiquitination and degradation 99. JQAD1 binds to p300 protein and target it for ubiquitin proteasome-mediated degradation 100. JQAD1 induces H3K27 deacetylation at core regulatory circuitry gene enhancers and reduces the transcription of critical oncogenes such as *MYCN* 100.

### CBP/p300 and BET bromodomain dual inhibitors

**Figure 2:**
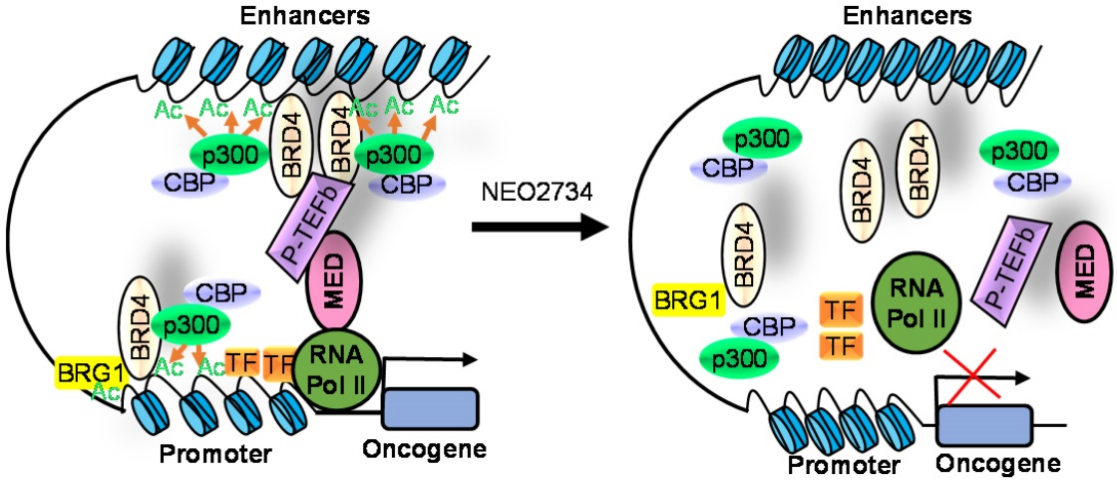
The CBP/p300 and BET bromodomain dual inhibitor NEO2734 suppresses H3K27 acetylation and oncogene transcription and expression. CBP/p300 induce H3K27 acetylation and recruit the BET bromodomain protein BRD4 at oncogene promoters, typical enhancers and super-enhancers, leading to oncogene transcriptional activation and over-expression. Treatment with NEO2734 blocks H3K27 acetylation and displaces BRD4 at oncogene promoters, typical enhancers and super-enhancers, leading to oncogene transcriptional suppression.

Due to homology between CBP/p300 and BET bromodomains, CBP/p300 and BET bromodomain dual inhibitors, NEO2734 and NEO1132, have been designed 101. NEO2734 binds both CBP/p300 and BET proteins with dissociation constants of 19 nM for CBP, 31 nM for p300 and 6 nM for BRD4. In comparison, NEO1132 displays dissociation constants of 61 nM for CBP, 80 nM for p300 and 63 nM for BRD4 102. NEO2734 downregulates the transcription of MYC target genes and genes involved in chemokine signalling and inflammation (Table 3) (Figure 2).

### CBP/p300 inhibitors as novel anti-cancer agents

The CBP/p300 HAT inhibitor C646 down-regulates oncogene expression, reduces acute myeloid leukemia cell clonogenic potential, and induces acute myeloid leukemia cell cycle arrest, apoptosis and melanoma and non-small cell lung cancer cell growth arrest 71, 74, 89 (Table 2). Consistent with these findings, the CBP/p300 bromodomain inhibitor I-CBP112 substantially reduces leukemic cell colony formation, induces leukemia cell differentiation, and suppresses leukemia-initiating potential of acute myeloid leukemia cells *in vitro* and in mouse models 96 (Table 3).

The CBP/p300 HAT inhibitor A-485 shows potent anticancer activity against acute myeloid leukaemia, multiple myeloma and non-Hodgkin's lymphoma cells 90. A-485 reduces H3K27ac at the enhancers of estrogen receptor α target genes, including *MYC* and *CCND1*, down-regulates their expression, and inhibits breast cancer cell proliferation. A-485 and the CBP/p300 bromodomain inhibitors CBP30, GNE-049 and CCS1477 reduce histone acetylation, suppress androgen receptor target gene transcription, and induce considerable anticancer effects against castration-resistant prostate cancer in mouse models 67, 72, 90, 97 (Tables 2 and 3).

In NUT midline carcinoma cells, A-485 reduces histone acetylation, disrupts BRD4-NUT binding, and down-regulates the expression of BRD4-NUT target genes including *MYC* and *TP63*. A-485 thereby strongly induces NUT midline carcinoma cell differentiation, cell cycle arrest and apoptosis 103 (Table 2).

The CBP/p300 HAT inhibitor B026 down-regulates *MYC* oncogene expression, and blocks acute myeloid leukemia cell proliferation *in vitro* and leukemia progression *in vivo* 91. B029-2 decreases amino acid metabolism and nucleotide synthesis gene expression, reduces glycolytic function and nucleotide synthesis, and suppresses hepatocellular carcinoma cell proliferation, migration and invasion *in vitro* and tumor progression *in vivo* 92 (Table 2).

In human neuroblastoma tissues, high levels of p300 expression correlates with poor patient prognosis, independent of current prognostic markers 100. The PROTAC compound JQAD1 selectively targets p300 protein for degradation, reduces the transcription of critical oncogenes such as *MYCN*, and induces neuroblastoma cell apoptosis *in vitro* and tumor growth inhibition in mouse models 100.

CBP/p300 inhibitors can also suppress tumor progression by activating immune response. Treatment with the CBP/p300 bromodomain inhibitor GNE-781 converts myeloid-derived suppressor cells from a suppressive to an inflammatory phenotype, decreases myeloid-derived suppressor cell differentiation and function, and impair *FOXP3* expression and T regulatory cell function. GNE-781 therefore augments tumor immune response and suppresses tumor growth in mouse models of colon and breast cancers 20, 98 (Table 3).

## CBP/p300 inhibitors exert synergistic anticancer effects with other anticancer agents

### Synergistic anticancer effects between CBP/p300 inhibitors and chemotherapy, radiotherapy and epigenetic therapy agents

CBP/p300 inhibitors exert synergistic anticancer effects with chemotherapy, radiotherapy and epigenetic therapy agents. Treatment with I-CBP112 decreases the expression of a number of ATP-binding cassette transporter genes in breast, lung and hepatic cancer cell lines, leading to increased accumulation of chemotherapy agents including daunorubicin, doxorubicin and methotrexate inside the cancer cells. I-CBP112 thereby considerably sensitizes cancer cells to a wide range of chemotherapy agents 96, 104. The CBP/p300 HAT inhibitor C646 sensitizes non-small cell lung cancer cells to radiotherapy by abrogating checkpoint maintenance and augmenting radiotherapy-induced mitotic catastrophe 105.

The DNA demethylation agent azacitidine is the best treatment for severe myelodysplastic syndromes, but patients eventually develop resistance. A loss of function shRNA screen identifies CBP as a top regulator of azacytidine resistance 76. In myelodysplastic syndrome-derived acute myeloid leukemia cells, CBP/p300 promote the expression of genes important for protein synthesis, and the CBP/p300 HAT inhibitor A-485 and the CBP/p300 bromodomain inhibitor CCS1477 synergize with azacytidine to induce myelodysplastic syndrome-derived leukemia cell death by synergistically suppressing global protein synthesis 76.

*EP300* gene transcription is up-regulated by EZH2 inhibitors, p300 drives oncogenic transcriptional reprograming and cancer cell resistance to EZH2 inhibitors, and C646 sensitizes diffuse large B-cell lymphoma cells to EZH2 inhibitors 106. In addition, A-485 induces H3K27 deacetylation and exerts synergistic anticancer effects with the histone demethylase KDM6A inhibitor GSK-J4 against multiple myeloma 20.

Interestingly, CBP/p300 HAT inhibitors and bromodomain inhibitors exert synergistic anticancer effects with each other and with BET bromodomain inhibitors. A-485 and I-CBP112 co-operatively reduce CBP/p300 occupancy at chromatin and the expression of androgen-dependent oncogenes such as *MYC* and *PSA*, and synergistically suppress prostate cancer cell proliferation 107. NUT midline carcinoma is characterized by NUT gene rearrangement with the BET bromodomain gene BRD4. Screening of an epigenetic compound library has identified the CBP/p300 HAT inhibitor A-485 and the BET bromodomain inhibitor JQ1 as the most effective in inducing NUT midline carcinoma cell growth inhibition and cell death 103. A-485 suppresses p300-mediated histone acetylation and the transcription of key oncogenes such as MYC; and combination therapy with A-485 and JQ1 synergistically suppresses Wnt/β catenin pathway and c-Myc target gene expression, and synergistically induces NUT midline carcinoma cell apoptosis 103. Similarly, I-CBP112 and the BET bromodomain inhibitor JQ1 synergistically induce leukemia cell apoptosis 96.

### Anticancer efficacy of the CBP/p300 and BET bromodomain dual inhibitors

In a panel of 60 cancer cell lines from different cancers, NEO1132 and NEO2734 display anti-proliferative effects in a number of cancer cell lines, with the most potent efficacy in leukemia, lymphoma and prostate cancer cells 102. NEO1132 and NEO2734 efficiently induce acute myeloid leukemia cell apoptosis, eliminates leukemic stem/progenitor cells, and enhance the anticancer effects of chemotherapies in mouse models of acute myeloid leukemia 108. NEO2374 induces transcriptional changes in lymphoma cells, significantly suppresses lymphoma progression in mice, and is more effective than CBP/p300 inhibitors or BET bromodomain alone 102.

In castration-resistant prostate cancer cells, treatment with NEO1132 or NEO2734 reduces H3K27ac at androgen receptor binding site-gained cell lineage and cancer-promoting gene loci, down-regulates their expression, induces castration-resistant prostate cancer cell growth inhibition and cell death *in vitro,* and abrogates tumors in mouse models 109.

### CBP/p300 inhibitors in clinical trials

Two CBP/p300 inhibitors are currently in clinical trials in cancer patients. CCS1477 is the first p300/CBP inhibitor in phase I/IIa clinical trials in patients with advanced solid tumours, such as *MYC* over-expressing small cell lung cancer, radiation-induced breast sarcoma and metastatic castrate-resistant prostate cancer (ClinicalTrials.gov Identifier: NCT03568656) 110. CCS1477 has also been evaluated in an open-label Phase I/IIa study as monotherapy in patients with advanced haematological malignancies including acute myeloid leukemia, non-Hodgkin lymphoma and multiple myeloma (ClinicalTrials.gov Identifier NCT04068597).

While no published pre-clinical information is available, the orally active, potent and selective CBP/p300 bromodomain inhibitor FT-7051 has entered a multi-center, phase I, open-label clinical trial in patients with metastatic castration-resistant prostate cancer (ClinicalTrials.gov Identifier NCT04575766) 111. Analysis of the first 5 enrolled patients has shown that FT-7051 causes minor side-effects including hyperglycemia 111.

## Conclusions and perspectives

The histone acetyltransferase CBP/p300 contains HAT domain and bromodomain which are required for H3K27 acetylation. CBP/p300-mediated H3K27ac is augmented by the histone “reader” BRD4, histone modification proteins, such as CBX, Trithorax, Trithorax-related proteins, UTX, MLL4 and SWI/SNF subunit proteins, transcription factors such as DUX4, the ubiquitin hydrolase USP24 and enhancer RNAs. CBP/p300 catalyzes histone H3K27ac at gene promoters, enhancers and super-enhancers, leading to transcriptional initiation and productive elongation. While CBP/p300 deletion/loss was known to promote tumorigenesis more than a decade ago, CBP/p300 have recently been demonstrated to be over-expressed or activated in cancer cells, induce enhancer and super-enhancer activity, and up-regulate the expression of important oncogenes, such as *MYC*, *MYCN*, *CCND1* and *ZNF395.* CBP/p300 therefore induce cancer cell proliferation, survival, migration, invasion, immune evasion, tumor initiation, progression and drug resistance in a number cancer types, such as leukemia, lymphoma, melanoma, hepatocellular carcinoma, lung and renal cancers.

It is currently not clear why CBP/p300 loss and over-expression/activity can both facilitate tumorigenesis. Possible explanations include: (i) CBP/p300 loss due to genetic deletion or rearrangement reduces the expression of tumor suppressor genes, the promoters of which are bound by CBP/p300; while CBP/p300 over-expression/activity leads to enhancer and super-enhancer formation at critical oncogene loci, leading to oncogene over-expression; (ii) cellular context and different expression levels of transcriptional co-factors alter CBP/p300 protein accessibility to oncogenic and tumor suppressive target gene loci; and (iii) CBP loss leads to over-activity of p300 and oncogene over-expression.

Originally regarded as a histone deacetylase inhibitor, the anticancer agent butyrate has now been confirmed to be CBP/p300 HAT activator. CBP/p300 HAT inhibitors and bromodomain inhibitors have been discovered through compound library screen, *in silico* compound screen and chemical synthesis. Among them, the CBP/p300 HAT inhibitors A-485, B026 and B029-2, and the bromodomain inhibitors GNE-049, GNE-781 and CCS1477 suppress CBP/p300 at < 10 nM. Other promising CBP/p300 targeting compounds include the PROTAC compound JQAD1 which induces p300 protein degradation, and the CBP/p300 and BET bromodomain dual inhibitor NEO2734. These CBP/p300 inhibitors and degrader reduce H3K27ac at oncogene promoters, enhancers and super-enhancer, down-regulate oncogene expression, induce cancer cell growth inhibition and apoptosis, suppress cancer cell migration, invasion, immune evasion and tumor progression. In addition, the CBP/p300 inhibitors exert synergistic anticancer effects with other anticancer agents such as chemotherapy, radiotherapy, EZH2 inhibitors and BET bromodomain inhibitors. Importantly, the CBP/p300 inhibitors CCS147 and FT-7051 are now in clinical trials in cancer patients.

While small molecule CBP/p300 HAT and bromodomain inhibitors have been the main focus of drug discovery research, the first PROTAC p300 protein degrader JQAD1 has recently been reported. Due to high homology between CBP and p300, current CBP/p300 HAT and bromodomain inhibitors suppress both of the enzymes with similar efficiency. In comparison, the PROTAC p300 degrader JQAD1 shows strong selectivity for p300 100. For clinical translation, CBP/p300 HAT and bromodomain inhibitors are expected to be preferred when both CBP and p300 are involved in tumorigenesis, such as prostate cancer with the over-expression of both CBP and p300 67, while PROTAC CBP or p300 protein degraders are required when only CBP or p300 is the tumorigenic driver, such as in *CBP*-deficient but p300 active lung cancer 74 as well as CBP non-functional but p300 active *MYCN* gene-amplified neuroblastoma 100.

Future endeavours should focus on developing more potent and selective small-molecule CBP/p300 HAT inhibitors, CBP/p300 and BET bromodomain co-inhibitors, and PROTAC CBP or p300 selective protein degraders through structure-based virtual screen, laboratory-based screen of small molecule compound libraries and chemical synthesis. Their safety to normal cells, pharmacokinetics and anticancer efficacy should be investigated in vitro and in mouse models. The other anticancer agent which exerts the best synergistic anticancer effects with CBP/p300 inhibitors or protein degraders can then be identified through approved oncology drug library screen. Ultimately, ideal combination therapies with CBP/p300 inhibitors or degraders and other anticancer drugs are expected to be tested in patients with cancers characterized by CBP and/or p300 over-expression or over-activity, and potential side-effects from the CBP/p300 inhibitors, degraders or combination therapies will be identified.

## Figures and Tables

**Table 1:** CBP/p300 induce oncogene transcription, cancer cell proliferation, survival, tumor initiation, tumor progression, metastasis and immune evasion

Cancer type	Modulation of gene expression	Modulation of tumorigenesis	References
Liver cancer	*EP300* gene is amplified/gained, reprograms super-enhancers, and up-regulates *MYC* and *CCND1*	Hepatocellular carcinoma cell proliferation *in vitro* and tumor progression in mice	66
Prostate cancer	CBP and p300 bind to androgen receptor-binding sites, and activate *MYC* transcription	Prostate cancer cell proliferation and androgen deprivation therapy resistance	67
Melanoma	In melanoma cells, p300 increases *MITF* and *FOXM1* gene transcription	Melanoma cell proliferation	68
Clear cell renal cell carcinoma	In clear cell renal cell carcinoma, *VHL* loss leads to p300 to *MYC* and *ZNF395* gene super-enhancers, and *MYC* and *ZNF395* over-expression	Clear cell renal cell carcinoma cell proliferation, survival, and colony formation *in vitro* and tumor progression in mice	69
Acute lymphoblastic leukemia	CBP induces super-enhancer formation at MYB binding sites, leading to *TAL1* over-expression	T-cell acute lymphoblastic leukemia cell survival and leukemogenesis	70
Acute myeloid leukemia	CBP/p300 modulate the transcription of genes involved in DNA replication and repair, mitosis and cell cycle progression	Leukemia cell immortalization, cell proliferation, survival and leukemia initiation and maintenance in mice	57, 71
Chronic myeloid leukemia & lymphoma	P300 binds to the *GATA1* and *MYC* gene super-enhancers and stimulate their over-expression	Chronic myeloid leukemia and lymphoma cell cycle progression and cell proliferation.	72
Diffuse large B-cell lymphoma	In diffuse large B-cell lymphoma with C-terminal truncated p300, the truncated p300 suppresses NF-κB and REL activity, reduces p53	Diffuse large B-cell lymphoma cell proliferation	37, 73
*CBP*- deficient lung cancer	In *CBP*-deficient lung cancer cells, p300 up-regulates *MYC* expression	Lung cancer cell cycle progression, proliferation, survival and tumor progression in mice.	74
Non-small cell lung cancer	P300 up-regulates IL-6, increases mesenchymal markers and decreases epithelial markers	Non-small cell lung cancer cell migration, invasion and metastasis	15, 75
Immune cells	In T regulatory cells and myeloid-derived suppressor cells, CBP/p300 up-regulates the expression of STAT pathway genes,* FOXP3* and *GATA3*	Enhance T regulatory cell and myeloid-derived suppressor cell function and survival; and suppress cytotoxic T cell-driven immunity, lymphocyte activation and proliferation	77-80

**Table 2:** Small molecule CBP/p300 HAT inhibitors and their anticancer effects

Compounds	Structures	HAT inhibition and anticancer effects	References
Lys-CoA	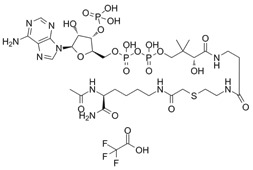	Suppresses p300-mediated histone acetylation with an IC_50_ of 0.5µM.	86
CCT077791	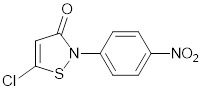	Reduces histone H3 and H4 acetylation and induces colon cancer cell growth inhibition with an IC_50_ of 2-3µM.	87
CCT077792	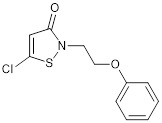	Reduces histone H3 and H4 acetylation and induces colon cancer cell growth inhibition with an IC_50_ of 0.4 µM.	87
C646	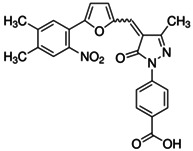	Suppresses p300-induced HAT activity with an inhibitory constant of 400 nM, reduces oncogene expression, induces melanoma, non-small cell lung cancer and acute myeloid leukemia cell cycle arrest and apoptosis, and sensitizes lymphoma cells to EZH2 inhibitors.	71, 74, 89, 106
A-485	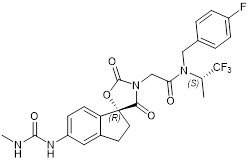	Suppresses CBP (IC_50_ = 2.6 nM) and p300 (IC_50_ = 9.8 nM), reduces estrogen receptor, androgen receptor and *MYC* gene expression, and show anticancer effects against breast, prostate, leukaemia, myeloma, lymphoma and NUT midline carcinoma.	67, 90, 97, 103
B026	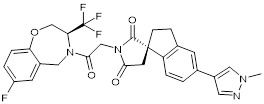	Suppresses CBP (IC_50_ = 9.5nM) and p300 (IC_50_ = 1.8nM), reduces *MYC* transcription, and blocks acute myeloid leukemia cell proliferation *in vitro* and leukemia progression *in vivo.*	91
B029-2	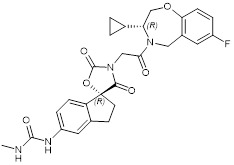	Suppresses CBP (IC_50_ = 11nM) and p300 (IC_50_ = 0.5nM), decreases amino acid metabolism and nucleotide synthesis gene expression, and reduces liver cancer cell proliferation *in vitro* and tumor progression *in vivo*.	92

**Table 3:** Small molecule CBP/p300 bromodomain inhibitors and their anticancer effects

Compounds	Structures	Bromodomain inhibition and anticancer effects	References
CPI703	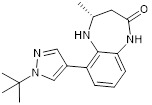	Suppresses CBP with an IC_50_ of 0.47 μM and cellular EC_50_ of 2.1 μM, reduces *FOXP3* transcription in regulatory T cells, and suppresses regulatory T cell differentiation and T helper 17 cell cytokine production.	93
CPI644	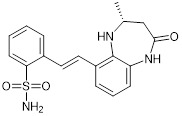	Inhibits CBP bromodomain with an IC_50_ of 0.18 μM and cellular EC_50_ values of 0.53 μM, and reduces the percentage of FOXP3(+) cells in differentiating regulatory T cells.	93
CBP30	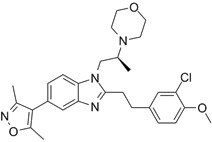	Suppresses CBP (IC_50_ = 21 nM) and p300 (IC_50_ = 38 nM), reduces IL-17A expression in immune cells and secretion by T helper 17 cells, and inhibits *MYC* and androgen target gene expression and prostate cancer cell proliferation.	72, 94
I-CBP112	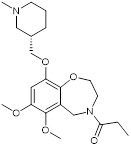	Inhibits CBP/p300 with dissociation constants of 151 nM for CBP and 167 nM for p300 and IC_50_ of 142 nM for CBP and 625 nM for p300, reduces immune response and drug resistance genes, and inhibits leukemia cell differentiation and leukemia-initiating potential in mice.	96, 104
GNE-049	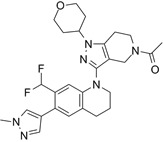	Suppresses CBP (IC_50_ = 1.1 nM) and p300 (IC_50_ = 2.3 nM), and represses the expression of oncogenes, such as *MYC* (EC_50_ = 14 nM) and androgen receptor target gene transcription, and induce anticancer effects against castration-resistant prostate cancer in mice.	97
GNE-781	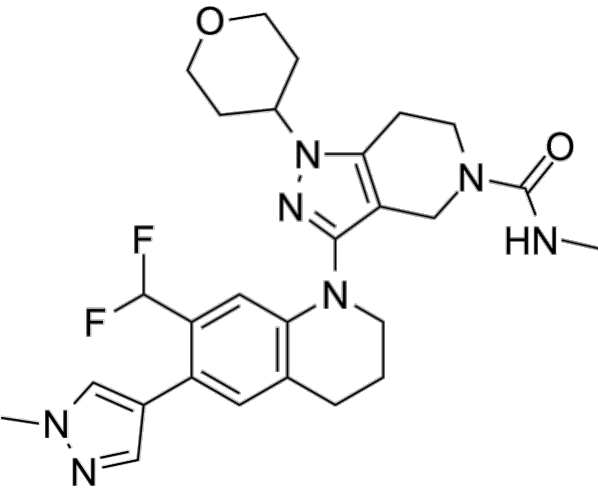	Suppresses CBP (IC_50_ = 0.94 nM) and p300 (IC_50_ = 1.2 nM), reduces *MYC* and *FOXP3* expression, decreases myeloid-derived suppressor and T regulatory cell function, augments tumor immune response, and suppresses colon and breast cancer in mice.	20, 98
CCS1477	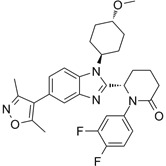	Binds to CBP and p300 (dissociation constants of 1.7 nM for CBP and 1.3 nM for p300; IC_50_ =19 nM), reduces androgen receptor coactivator function and androgen target gene transcription, and induce anticancer effects against castration-resistant prostate cancer. Synergizes with azacytidine to induce leukemia cell death.	67, 76
NEO2734	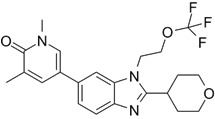	Shows dissociation constants of 19 nM for CBP, 31 nM for p300 and 6 nM for BRD4, reduces the transcription of MYC target genes and genes involved in chemokine signalling and inflammation, and reduces leukemia, lymphoma and prostate cancer growth *in vitro* and *in vivo*.	102, 108, 109

## Abbreviations

AIL: autoinhibitory loop
CBP: CREB-binding protein
CH2: cysteine/histidine-rich region
HAT: histone acetyltransferase
H3K27: histone H3 lysine 27
H3K27ac: histone H3 lysine 27 acetylation
IBiD: interferon-binding domain
IC_50_: half-maximal inhibitory concentration
Janus kinase: JAK
KIX: kinase inducible domain of CREB interacting domain
mitogen-activated protein: MAP
NRID: nuclear receptor interaction domain
PCAF: p300/CBP associated factor
PROTAC: proteolysis-targeted-chimaera
P-TEFb: positive transcription elongation factor b
RNA Pol: RNA polymerase
RNA Pol II: RNA polymerase II
signal transducer and activator of transcription: STAT
TAZ1: transcriptional-adaptor zinc-finger domain 1
TAZ2: transcriptional-adaptor zinc-finger domain 2
ZZ: ZZ-type zinc finger domain
